# Flogging a dead horse

**DOI:** 10.1186/2041-2223-4-5

**Published:** 2013-02-28

**Authors:** Mark A Jobling

**Affiliations:** 1 Department of Genetics, University of Leicester, University Road, Leicester, LE1 7RH, UK

## 

We may live in a global village, and do our research in multinational departments, but local cultural traditions still count. A chilly October morning in the bleak English Midlands, and a PhD student newly arrived from Italy (let’s call her Giovanna) takes a cigarette break outside our building. In the camaraderie of smokers, she strikes up a conversation with Keith, who is taking a rest from the autoclaving. “Where can I find horse?”, she asks. Processing this odd question, via her foreign-ness, he pictures Leicester race-course, and wonders about country riding clubs. But he is wide of the mark. Giovanna’s thoughts are in the kitchen, and when Keith realizes this, the conversation stops short.

For the British (and for most of the English-speaking world), eating horsemeat (hippophagy) is just not an option, something they would never, ever do. At least, that’s what we all thought until recently, when it turned out that many of us had been unwittingly doing exactly that for some time. The ‘horsemeat crisis’ has moved from the detection of a few percent contamination in burgers to the finding of lasagne purportedly made with beef, but actually being pure, unadulterated horse. The health risks are probably minimal – there is some concern about a drug, phenylbutazone, allowed in horses but prohibited in humans – but nonetheless many tonnes of processed foods have been destroyed.

According to Alan Davidson’s compendious and wonderful *Oxford Companion to Food*[1], the most avid consumers of horsemeat are the Italians, particularly those from the region around Venice, but there is also considerable enthusiasm among the peoples of France, Belgium, the Netherlands, Germany, Sweden and Iceland. A dislike of horsemeat is one of those things (along with a similar feeling about frogs and snails) that distinguishes a true Brit from a Frenchman – what some have called ‘gastro-nationalism’. To the novice British tourist in France a *boucherie chevaline*, the specialist horse-butcher marked by a gilded horse-head above the door, is a surprising sight. Interestingly, given the current crisis, the reason that these were kept distinct from other butchers (*bouchers classiques*) was to guard against the threat of fraudulent sale of horse as beef.

Revulsion at eating horses is partly connected to the idea of the horse as a noble animal, but also to the more pragmatic point that, as a means of transport and traction, it was traditionally worth more alive than dead. In medieval times eating horses was a desperate act associated with famine, so a link was formed between hippophagy and poverty. Nineteenth-century attempts to turn British attitudes around [2] included a proposal to rename horsemeat *cheval*, in the same euphemistic way that we use words derived from French terms for the meat of the pig (*porc*) and cow (*boeuf*). In 1868 a horse banquet was held for 150 guests by London’s *Society for the Propagation of Horse Flesh as an Article of Food.* Despite all this, it didn’t catch on. Apart from the general dislike, a particular problem may have been that British horsemeat came from tough old animals at the end of their working lives.

Finding the horse in your burger requires DNA analysis, and exploits the marked interspecies differences between mitochondrial DNAs (mtDNAs) that result from their relatively high mutation rates. The properties that have made this molecule a popular tool in animal species ‘barcoding’ projects [3] also allow the design of real-time polymerase chain reaction (PCR) assays that specifically identify horse DNA in a mixture of meats from other species. These assays are remarkably sensitive, allowing the detection of levels as low as 0.0001% [4]. The journal *Meat Science* has plenty of articles on this topic, including a nice sausage-specific example [5].

Such studies also describe the identification of a wide range of species mixtures other than horse and cow, including chicken, turkey, sheep, pig, and donkey. Distinguishing horse from donkey can be tricky, as the species are closely related, but apparently important, because some cultures eat one, but not the other. The Italians are fond of both.

However, as well as the sausage manufacturers, horses (*Equus caballus*) and donkeys (*Equus asinus*) can do the mixing themselves, to produce interspecific hybrids - mules and hinnies. The nature of the offspring depends on the sex of the parents, and a mnemonic helps here: for a mule, the mare is the mother; for a hinny, the he is a horse. Producing mules (the more common and useful hybrid) is not an easy business, because, ‘in equine courtship it is the stallion who takes the initiative, and… the donkey stallion is a fastidious fellow, and he does not willingly engage in anything which might be regarded as slightly improper’ [6]. The phenotypes are different (probably as a result of parentally imprinted genes), with a mule appearing to have a donkey’s ears and a horse’s tail, and a hinny the other way round. People eat mules, as well as donkeys and horses, and in meat contamination testing, mule meat would appear to be horsemeat, because of the maternal inheritance of mtDNA.

Horses have 64 chromosomes, and donkeys 62, so mules and hinnies carry the intermediate odd number 63, which leads to infertility in the hybrids because oocytes fail during meiosis. As Thomas Bewick writes in his *A General History of Quadrupeds*[7], ‘Nature has providently stopped the further propagation of these heterogeneous productions, to preserve, uncontaminated, the form of each animal; without which, …every creature, losing its original perfection, would rapidly degenerate’. Occasionally, though, a female mule produces a foal after mating with a male donkey; so rare is this event that the Romans used the phrase *Cum mula peperit* (when a mule gives birth), equivalent to the English ‘once in a blue moon’. One such product, a healthy female in China who could plough a field by herself at 4 years of age, had 62 chromosomes, a mix of horse and donkey with a bias towards the latter [8].

In the global market-place of the internet, obtaining novel and exotic animal food products is becoming ever easier, and if you have a strong stomach and wonder how broad the scope is, just visit http://www.exoticmeatmarkets.com, where you’ll find pretty much everything (except horse…). But are these things really what they say? Contributors to *Meat Science*, take note – there is work for you to do!

Back in our multinational department, too, the gastro-exotica goes on, as my Estonian post-doc kindly brings me a present: a tin of *karuliha* – bear-meat. We have yet to eat it.

## Competing interests

The author declares that he has no competing interests.
